# Pharmacologic Antagonization of Cannabinoid Receptor 1 Improves Cholestasis in *Abcb4*^*-/-*^ Mice

**DOI:** 10.1016/j.jcmgh.2021.12.013

**Published:** 2021-12-23

**Authors:** Nora Helmrich, Martin Roderfeld, Anne Baier, Anita Windhorst, Diran Herebian, Ertan Mayatepek, Christian Dierkes, Matthias Ocker, Dieter Glebe, Bruno Christ, Yuri Churin, Karuna Irungbam, Elke Roeb

**Affiliations:** 1 Department of Gastroenterology, Giessen, Germany; 2 Institute for Medical Informatics, Justus Liebig University, Giessen, Germany; 3 Department of General Pediatrics, Neonatology and Pediatric Cardiology, Medical Faculty, University Hospital Duesseldorf, Heinrich Heine University, Duesseldorf, Germany; 4 Medizinisches Versorgungszentrum for Pathology, Justus Liebig University Giessen, Trier, Germany; 5 Institute for Surgical Research, Philipps University of Marburg, Marburg, Germany; 6 Institute of Medical Virology, National Reference Centre for Hepatitis B Viruses and Hepatitis D Viruses, Justus Liebig University, Giessen, Germany; 7 Applied Molecular Hepatology Laboratory, Department of Visceral, Transplant, Thoracic and Vascular Surgery, University of Leipzig Medical Center, Leipzig, Germany

**Keywords:** Liver, Rimonabant, Bile Acid, Acute Phase, Fibrosis, ACEA, arachidonyl-2'-chloroethylamide, ALT, alanine aminotransferase, AST, aspartate aminotransferase, c-JUN, cellular JUN, CB, cannabinoid receptor, CD45, cluster of differentiation antigen 45, CK19, cytokeratin 19, ECM, extracellular matrix, FASN, fatty acid synthase, JNK, c-Jun N-terminal kinase, LCN2, lipocalin 2, mRNA, messenger RNA, PCK1, phosphoenolpyruvat carboxykinase, PLIN2, perilipin 2, PPAR, peroxisome proliferator activated receptor, qRT-PCR, quantitative reverse-transcription polymerase chain reaction, SREBP-1, sterol regulatory element-binding protein 1, STAT3, signal transducer and activator of transcription 3, WT, wild-type

## Abstract

**Background & Aims:**

The endocannabinoid system is involved in the modulation of inflammatory, fibrotic, metabolic, and carcinogenesis-associated signaling pathways via cannabinoid receptor (CB)1 and CB2. We hypothesized that the pharmacologic antagonization of CB1 receptor improves cholestasis in *Abcb4*^*-/-*^ mice.

**Methods:**

After weaning, male *Abcb4*^*-/-*^ mice were treated orally with rimonabant (a specific antagonist of CB1) or ACEA (an agonist of CB1) until up to 16 weeks of age. Liver tissue and serum were isolated and examined by means of serum analysis, quantitative real time polymerase chain reaction, Western blot, immunohistochemistry, and enzyme function. Untreated *Abcb4*^*-/-*^ and Bagg Albino Mouse/c wild-type mice served as controls.

**Results:**

Cholestasis-induced symptoms such as liver damage, bile duct proliferation, and enhanced circulating bile acids were improved by CB1 antagonization. Rimonabant treatment also improved Phosphoenolpyruvat-Carboxykinase expression and reduced inflammation and the acute-phase response. The carcinogenesis-associated cellular-Jun N-terminal kinase/cellular-JUN and signal transducer and activator of transcription 3 signaling pathways activated in *Abcb4*^*-/-*^ mice were reduced to wild-type level by CB1 antagonization.

**Conclusions:**

We showed a protective effect of oral CB1 antagonization in chronic cholestasis using the established *Abcb4*^-*/-*^ model. Our results suggest that pharmacologic antagonization of the CB1 receptor could have a therapeutic benefit in cholestasis-associated metabolic changes, liver damage, inflammation, and carcinogenesis.

## Graphical abstract

**Figure:**
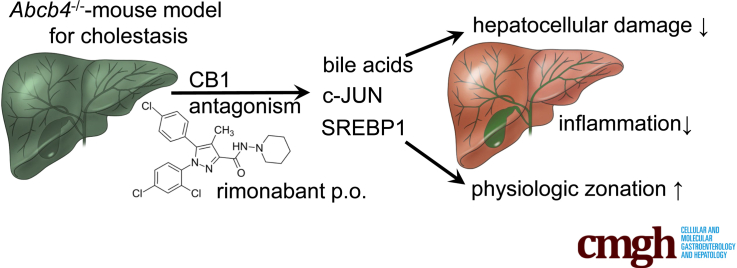

**Summary**

Antagonization of endocannabinoid receptor 1 by rimonabant ameliorated metabolic, inflammatory, and carcinogenesis-associated processes, as well as serum bile acids in *Abcb4*-knockout mice. This leads to an improvement of cholestasis and reduced hepatic pathogenesis.

Every year, more than 1.2 million people die from complications of cirrhosis, which currently is the 11th most common cause of death worldwide.^1^ This number shows the need to establish new and effective therapy options. After removing the cause, the damaged liver usually is able to restore its function, even in the presence of advanced cirrhosis. Until now, however, there has not been an effective pharmacologic antifibrotic therapy for advanced liver injury resulting from chronic cholestasis such as sclerosing cholangitis.

A large number of studies have shown that the endocannabinoid system is a significant mediator of acute and chronic liver disease. The function of the 2 cannabinoid receptors (CB)1 and CB2 has been a subject of scientific research for many years. In addition to their metabolic effects, new insights into their role in the development of liver inflammation and fibrosis in chronic liver damage are of great interest.^2^^,^^3^

Teixeira-Clerc et al^2^ were able to show that CB2 had anti-inflammatory and antifibrotic effects in the early stages of liver damage.This protective influence was eliminated by the profibrotic effects of activated CB1 if liver damage persisted.^4^ In chronically damaged liver tissue, CB1 is strongly expressed and stimulated by increased release of endocannabinoids by hepatocytes, hepatic stellate cells, and Kupffer cells. It shows its strongest expression in nonparenchymal cells such as inflammatory cells, proliferating cholangiocytes, hepatic stellate cells, and portal myofibroblasts.^2^^,^^4^

Rimonabant, also called SR141716A, belongs to the active ingredient class of anorectics and is a selective CB1 antagonist. The CB1 blockade in the diseased liver showed positive effects on the development of steatohepatitis, fibrosis, and the metabolic syndrome in several animal models.^2^^,^^5^^,^^6^

Recently, we showed that a global knockout of the *Cb1* gene (*Cb1*^*-/-*^) reduced the expression of the lipid droplet binding protein Perilipin 2 (PLIN2) in the livers of *Cb1*^*-/-*^ and hepatitis B surface protein–transgenic mice, which spontaneously develop hepatic steatosis. In addition, the antagonization of CB1 in human cell culture also caused a reduction of PLIN2, a cytoplasmic lipid droplet binding protein involved in the storage of neutral lipids within the lipid droplets.^7^

The CB1 receptor is down-regulated during cholestasis. Anandamide, a partial CB1 agonist, suppresses cholangiocyte growth in bile duct ligation (BDL) mice by induction of cholangiocyte apoptosis.^8^

*Abcb4*^*-/-*^ mice represent a well-characterized model for sclerosing cholangitis beginning with persistent cholestasis that progresses to cirrhosis and liver failure before late childhood.^9^ Although knowing the basic genetic defects and the pathology of the disease, being characterized by ductular proliferation in the liver and progressive intrahepatic cholestasis, there is still no successful therapeutic approach.^10^

In our present work, the effect of CB1 antagonization on cholestasis and its consequential damage was examined. For this purpose, *Abcb4*^*-/-*^ mice were treated with the selective CB1 antagonist rimonabant. Our hypothesis was that the disease progression and consequences of cholestasis can be delayed by CB1 antagonization. Metabolic parameters and specific markers for inflammation, fibrogenesis, and carcinogenesis were examined. Untreated *Abcb4*^*-/-*^ mice and mice fed with the CB1 agonist arachidonyl-2-chloroethylamide (ACEA) were included as controls.

## Results

### Cholestatic Liver Disease

**Figure 1:**
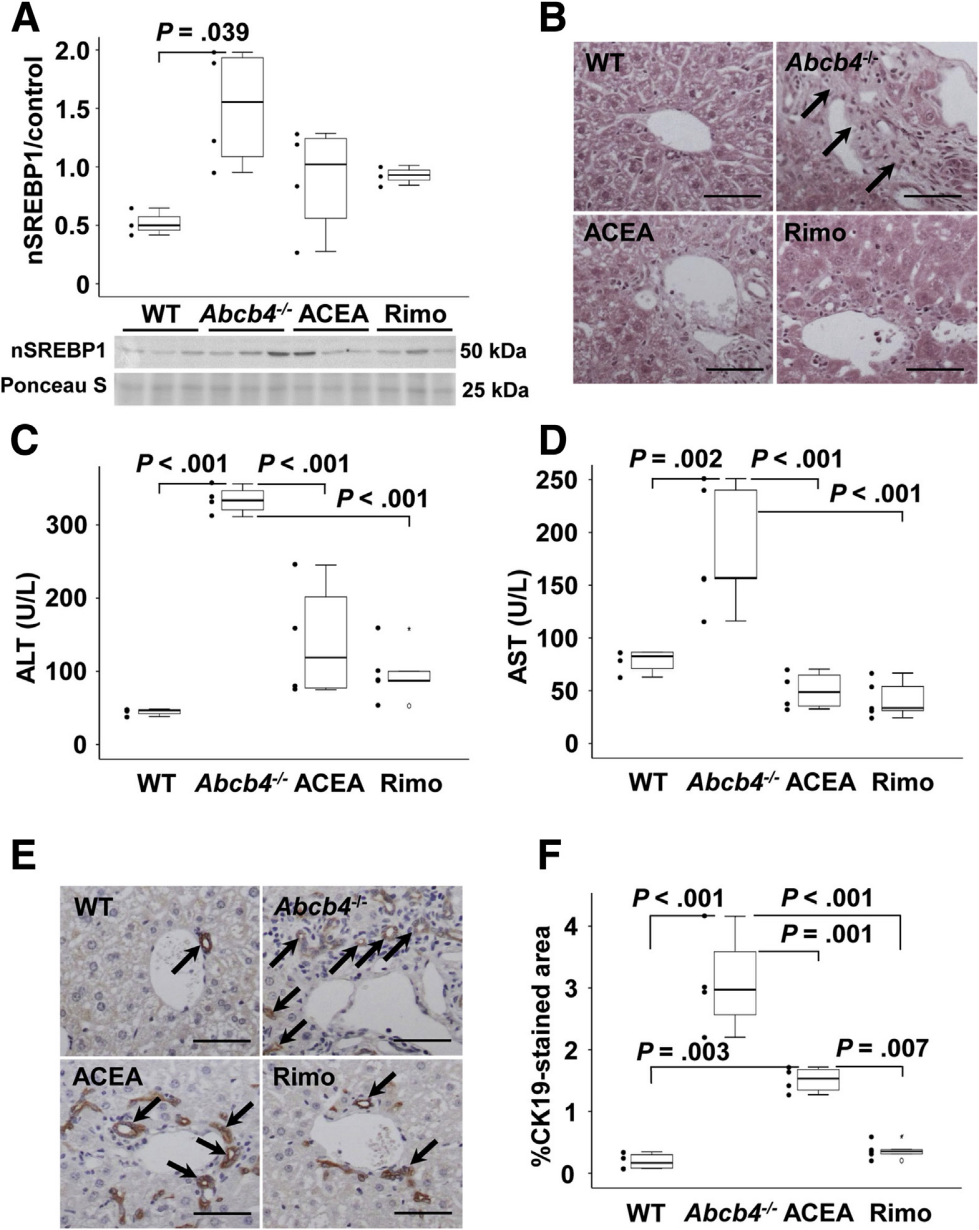
**Rimonabant and ACEA reduced cholestatic liver injury in *Abcb4***^**-/-**^**mice.** (*A*) Nuclear SREBP (nSREBP)1 was induced in *Abcb4*^-/-^ mice. Treatment with rimonabant or ACEA tendentially reduced hepatic nSREBP1. Densitometric analysis was performed with ImageJ software. n = 3–4 mice per group. Representative immunoblotting data of 3 independent experiments are shown. (*B*) H&E staining suggested that the cholestatic changes in the portal fields of the *Abcb4*^-/-^ mice were improved by treatment with ACEA and rimonabant. Original magnification: 200×. *Scale bars*: 100 μm. *Arrows* indicate cholestasis-induced periportal alterations of hepatic architecture. (*C* and *D*) ALT and AST serum levels were induced in *Abcb4*^-/-^ mice. CB1 antagonization by rimonabant but also treatment with ACEA caused a considerable reduction in serum levels of ALT and AST, indicating reduced hepatocellular injury. Serum aminotransferases were quantified in 2 independent experiments. n = 3–5 mice per group. (*E* and *F*) CK19-immunostaining (*arrows*) visualized enhanced ductular reaction in *Abcb4*^-/-^ mice. CK19 immunostaining showed reduced ductular proliferation in ACEA- and rimonabant-treated mice. The relative area that was stained for CK19 by immunohistochemistry was quantified using ImageJ. (*E*) Original magnification: 200×. *Scale bars*: 100 μm. (*A*, *C*, *D*, and *F*) One-way analysis of variance with a post hoc Bonferroni test was used for statistical analysis. Rimo, rimonabant.

The sterol regulatory element-binding protein-1 (SREBP1) plays a crucial role in the regulation of cholesterol metabolism and fatty acid synthesis. SREBP1 is a downstream effector of CB1, thus contributing to the development of obesity and fatty liver via lipogenesis.^11^ It has been shown that cholestasis was associated with reduced messenger RNA (mRNA) expression of *Srebp1* and diminished lipogenesis in *Abcb4*^*-/-*^ mice.^12^^,^^13^ Here, we isolated nuclear proteins and analyzed the nuclear amount of the matured transcription factor nuclear SREBP1. Interestingly, we found enhanced amounts of matured nuclear SREBP1 protein in *Abcb4*^*-/-*^ mice compared with wild-type (WT) mice, while neither rimonabant nor ACEA altered nuclear SREBP1 significantly (Figure 1*A*).

In the course of slowly progressing cholestasis, an increase in connective tissue remodeling was observed in the liver (black arrows, Figure 1*B*, upper right) of untreated *Abcb4*^*-/-*^ mice, and was most evident in the periportal fields (Figure 1*B*, upper right). The liver structure and the remodeling of connective tissue in mice treated with rimonabant (Figure 1*B*, lower right) was comparable with that of WT mice (Figure 1*B*, upper left). Remarkably, pathologic changes in the portal fields of the *Abcb4*^*-/-*^ mice also were reduced by treatment with ACEA (Figure 1*B*, lower left). Although histologic grading suggested improved scoring in rimonabant-treated animals, statistical significance was not reached.

In the course of hepatocellular damage, accompanying cholestatic disease alanine aminotransferase (ALT) (Figure 1*C*) and aspartate aminotransferase (AST) (Figure 1*D*) serum values increase. Serum levels of ALT and AST were reduced by treatment with both rimonabant and ACEA. There were no differences in serum ALT and AST levels between rimonabant- and ACEA-treated mice (Figure 1*C* and *D*).

Cytokeratin 19 (CK19) immunostaining (black arrows) visualized a higher number of periportal bile ducts in *Abcb4*^*-/-*^ mice and thus showed the highest level of bile duct proliferation in untreated *Abcb4*^*-/-*^ mice (Figure 1*E*, upper right). Rimonabant treatment and, to a lesser degree, ACEA treatment resulted in reduced bile duct proliferation (Figure 1*E* and *F*).

**Figure 2:**
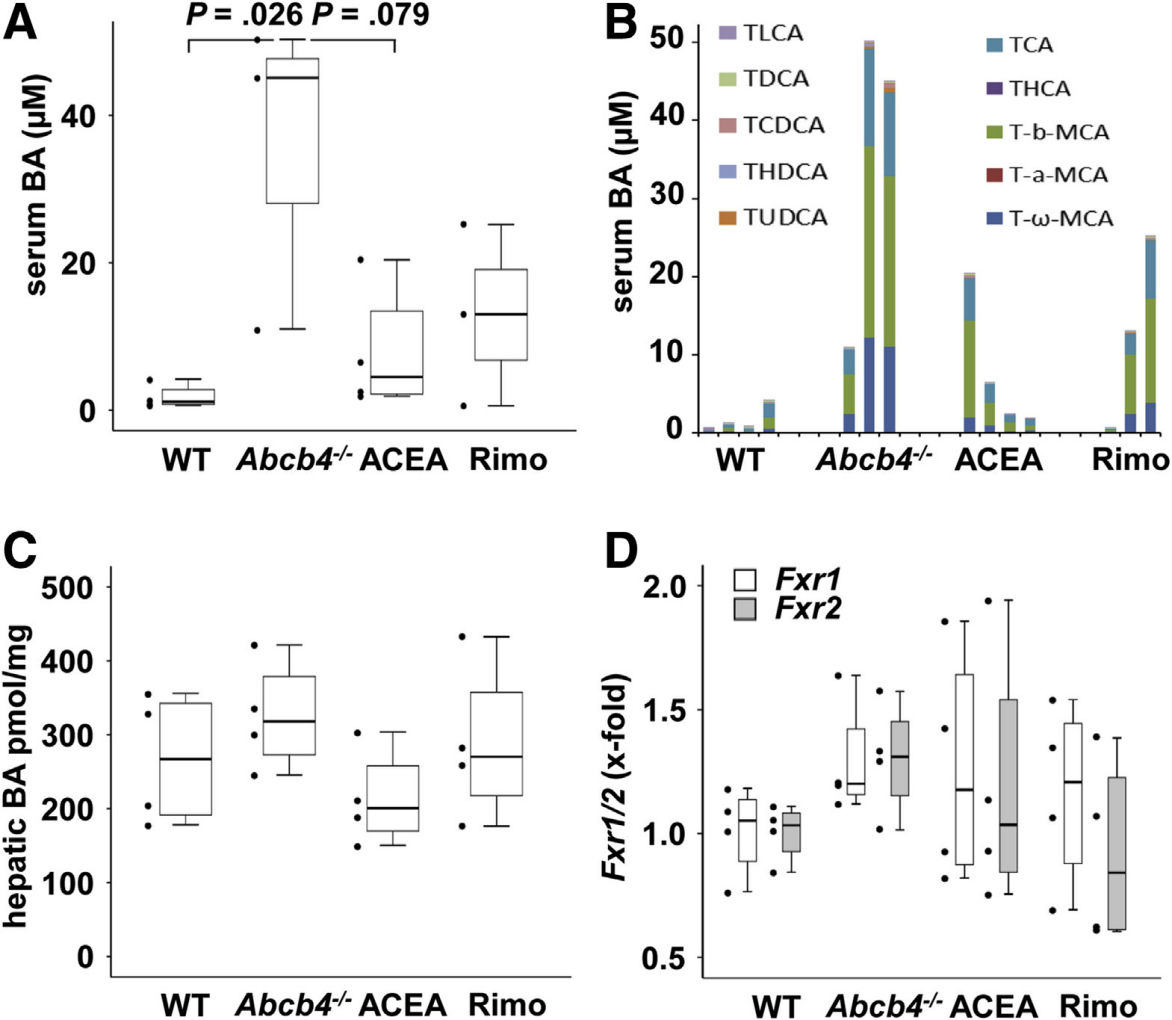
**Serum and hepatic bile acid levels.** (*A*) Total serum bile acid concentration was increased in *Abcb4*^-/-^ mice. ACEA and rimonabant treatment tendentially reduced serum bile acid concentrations. (*B*) Individual serum bile acid concentrations showed considerable interindividual differences within the groups. Serum bile acid quantification was performed once. (*C*) Interestingly, hepatic bile acid concentrations were not significantly different between the groups. (*D*) Quantitative real-time PCR showed equal amounts of hepatic mRNA levels of farnesoid X receptor (Fxr)1 and Fxr2 in all groups. BA, bile acids; MCA, muricholic acids; TCA, taurocholic acid; TCDCA, taurochenodeoxycholic acid; TDCA, Taurodeoxycholic acid; THCA, trihydroxycholestanoic acid; THDCA, taurohyodeoxycholic acid; TLCA, taurolithocholic acid; TUDCA, tauroursodeoxycholic acid.

To determine the degree of cholestasis quantitatively, serum bile acids were analyzed. Cholestasis, particularly because of *Abcb4*^*-/-*^, leads to a loss of barrier function of bile ducts caused by changes in the tight junctions. This consequently leads to an increase of serum bile acids (Figure 2*A*). ACEA and rimonabant tendentially reduced serum bile acid concentrations.

The bile acids transferred into the serum as part of the cholestatic disease showed a clear dominance of the taurine-conjugated bile acids T-ω-muricholic acid and T-β-muricholic acid in the serum of *Abcb4*^*-/-*^ mice. This predominance of taurine conjugates is typical in mice (Figure 2*B*). Interestingly, hepatic bile acid content as well as the hepatic expression of *Fxr* was not altered significantly, either in *Abcb4*^-/-^ mice or by treatment with ACEA or rimonabant (Figure 2*C* and *D*).

### Hepatic Metabolism

**Figure 3:**
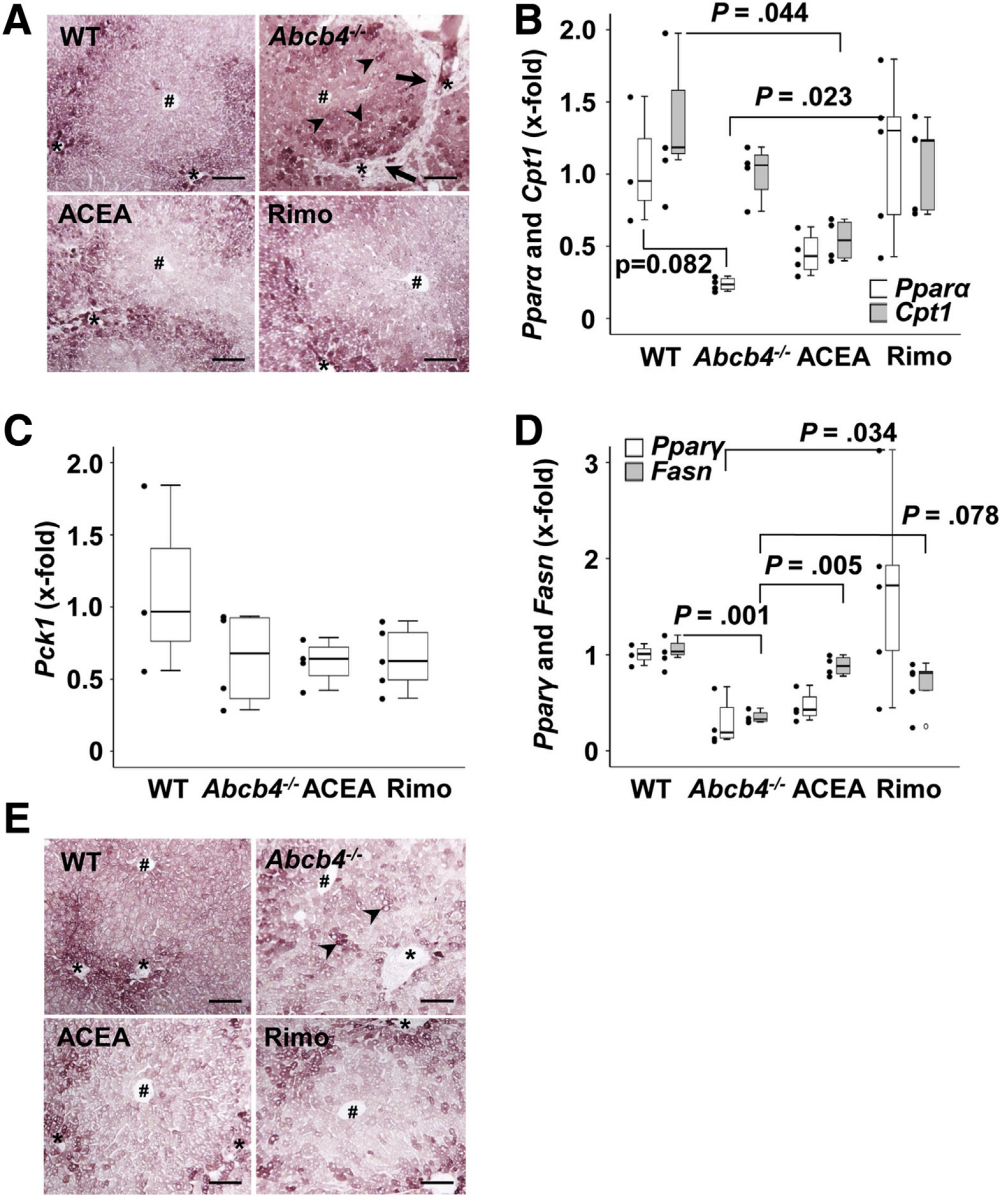
**Treatment with ACEA and rimonabant ameliorated cholestatic metabolic changes in *Abcb4***^**-/-**^**mice.** (*A*) Immunostaining showed periportal localization of PCK1 in healthy liver (*upper left panel*). Zonation of PCK1 expression was disrupted in *Abcb4*^-/-^ mice (*upper right panel*). Treatment with ACEA and rimonabant largely preserved the healthy zonation pattern of PCK1 (*lower panels*). *Arrowheads* indicate the disturbed zonal expression of PCK1 in *Abcb4*^-/-^ mice. *Arrows* indicate periportal fibroinflammatory infiltrates without PCK1 expression. (*B*) *Pparα* was tendentially reduced in *Abcb4*^-/-^ mice. Treatment with rimonabant normalized hepatic *Pparα* mRNA to healthy control levels. Interestingly, PPARα-regulated gene expression of *Cpt1* was not altered in *Abcb4*^-/-^ mice, but was reduced by treatment with ACEA. (*C*) *Pck1* mRNA levels were constant among all groups. (*D*) Hepatic *Pparγ* transcription was tendentially reduced in *Abcb4*^-/-^ mice. Rimonabant treatment increased *Pparγ*. *Fasn* mRNA was decreased significantly in cholestatic *Abcb4*^-/-^ liver. Treatment with ACEA normalized *Fasn*, while the induction of *Fasn* by rimonabant was not statistically significant. (*E*) Immunostaining of FASN depicted the zonated expression of FASN. Similar to the disturbed distribution of PCK1 (shown in panel *A*), the zonation of FASN also was disrupted in cholestatic liver of *Abcb4*^-/-^ mice and preserved by treatment with ACEA and rimonabant. (*B–D*) One-way analysis of variance and post hoc Bonferroni test were used for statistical analysis. n = 3–5 mice per group. Representative data of 1 of 3 independent experiments are shown. Original magnification: 100×. *Scale bars*: 100 μm. #Central vein, ∗portal tract. Rimo, rimonabant.

Cytosolic phosphoenolpyruvate carboxykinase (PCK1) catalyzes the conversion of oxaloacetate to phosphoenolpyruvate, the rate-determining step of gluconeogenesis. In the healthy liver, gluconeogenesis takes place mainly in periportal hepatocytes, where a higher expression of PCK1-positive cells can be displayed. A pathologic disturbance of the metabolic zonation in *Abcb4*^*-/-*^ mice was detected by immunohistochemical PCK1 staining. The liver tissue of untreated *Abcb4*^*-/-*^ mice showed an impaired structure (Figure 3*A*, arrows) and zonation (Figure 3*A*, arrowheads). Treatment with rimonabant largely preserved a clear zonation, similar to the WT controls. Intriguingly, treatment with ACEA also preserved zonation to a large extent, which might indicate additional physiologic relevance apart from CB1 agonization. The serum glucose levels remained unchanged in all groups (data not shown).

Peroxisome proliferator activated receptor (PPAR)α is involved in the regulation of lipid catabolism and glucose homeostasis. Gene expression of important regulators of glucose metabolism, such as *Pparα*, *Cpt1*, and *Pck1*, were examined by quantitative reverse-transcription polymerase chain reaction (qRT-PCR) at the mRNA level (Figure 3*B* and *C*).

*Pparα* but not *Cpt1* gene expression showed a tendential down-regulation in untreated *Abcb4*^*-/-*^ mice compared with WT mice (*P* = .082). Rimonabant treatment normalized *Ppar*α gene expression (*P* = .023), but had no effect on *Cpt1*. ACEA treatment had no effect on *Pparα* (Figure 3*B*). Although *Cpt1* was not altered in *Abcb4*^-/-^ mice, we found reduced expression in ACEA-treated *Abcb4*^-/-^ mice in comparison with WT mice (Figure 3*B*). In summary, zonation of PCK1 and gene expression of *Pparα* were normalized by rimonabant treatment. Serum glucose levels were largely unaffected by these changes. However, although zonal perturbation occurred, the total amount of hepatic *Pck1* mRNA did not change (Figure 3*C*).

Because glucose and lipid metabolism are closely linked, the gene expression of *Fasn* and the transcription factor *Pparγ* were examined, both of which have regulatory functions in lipid metabolism. The hepatic amount of *Fasn* mRNA was down-regulated in untreated *Abcb4*^*-/-*^ mice. Rimonabant treatment tendentially increased *Fasn*, while ACEA treatment normalized *Fasn* gene expression to WT levels (Figure 3*D*). The gene expression of *Pparγ* was not altered in *Abcb4*^*-/-*^ mice compared with WT mice. Rimonabant treatment led to an up-regulation, compared with untreated *Abcb4*^*-/-*^ mice (Figure 3*D*).

Immunohistochemical staining of fatty acid synthase (FASN) also showed a disturbed zonation (Figure 3*E*, arrowheads) and a preserved distribution of hepatocellular FASN protein expression after ACEA and rimonabant treatment.

In summary, a pathologic remodeling of the liver was found in the untreated *Abcb4*^*-/-*^ group with dissolved zonation and lobular structure. Analogous to the liver section of the WT group, the rimonabant-treated (and also ACEA-treated) group showed a clear zonation of the liver. Normal liver structure and enzyme values at the WT level were found in both treatment groups.

### Hepatic Inflammation

**Figure 4:**
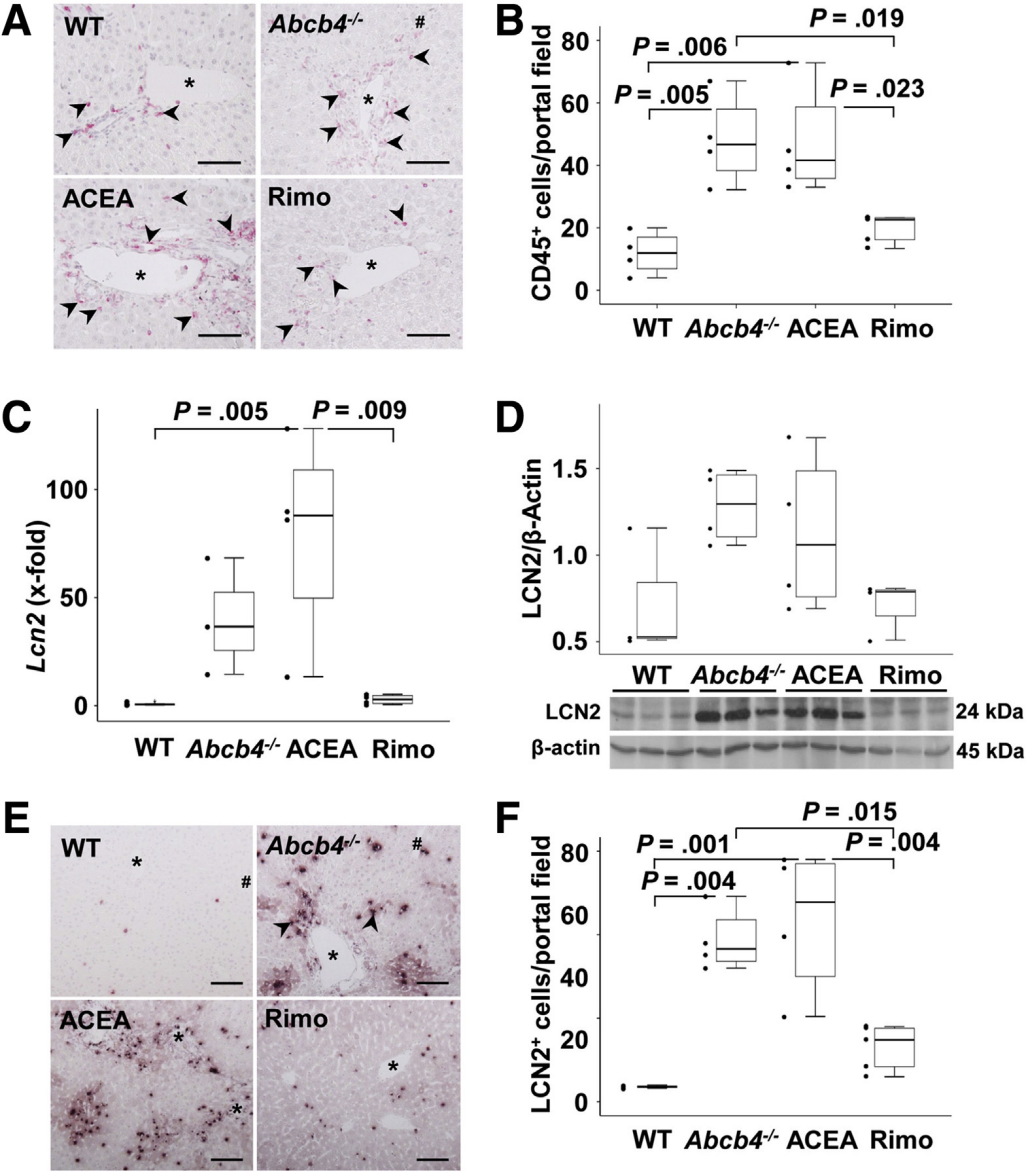
**Rimonabant reduced hepatic inflammation in *Abcb4***^**-/-**^**mice.** (*A* and *B*) Immunostaining of CD45 and counting of positive cells per portal field indicated enhanced numbers of CD45^+^ cells (*arrowheads*) infiltrated in the portal tracts of *Abcb4*^*-/-*^ and ACEA-treated mice, while treatment with rimonabant reduced the numbers of these cells. Original magnification: 200×. n = 4–5 mice per group. CD45^+^ cells in at least 5 randomly chosen portal tracts were counted. (*C*) Hepatic mRNA of *Lcn2* was enhanced in ACEA-treated mice and normalized to WT control levels by rimonabant treatment. (*D*) Western blot analysis suggested comparable protein regulation of LCN2 protein as observed on mRNA level (*C*). Nevertheless, densitometric and statistical analysis did not show significant differences between the groups. These are representative immunoblotting data of 2 independent experiments. (*E* and *F*) Immunohistochemistry showed enhanced numbers of LCN2-positive cells in *Abcb4*^*-/-*^ mice and reduced hepatic infiltration of LCN2-positive cells (*arrowheads*) in rimonabant-treated mice. Original magnification: 100×. (*B–D* and *F*) One-way analysis of variance and post hoc Bonferroni tests were used for statistical analysis. n = 3–5 mice per group. One of 3 independent experiments of mRNA analyses is shown. *Scale bars*: 100 μm. ∗Portal vein. Rimo, rimonabant.

Former studies showed that the alterations in lipid metabolism mediate inflammation, fibrosis, and proliferation in *Abcb4*^*-/-*^ mice.^12^ Immunostaining showed an infiltration of a higher number of cluster of differentiation antigen 45 (CD45)-positive leukocytes in untreated and ACEA-treated Abcb4^-/-^ mice (Figure 4*A* and *B*). Remarkably, the treatment with rimonabant reduced this cholestasis-associated infiltration of inflammatory cells. Histopathologic examination also suggested a lower hepatic inflammation score in the rimonabant-treated group, but statistical significance was not reached. Likewise, the H&E staining might indicate a moderate infiltration of inflammatory cells in this group (Figure 1*B*).

Lipocalin-2 (LCN2, also named neutrophil gelatinase-associated lipocalin (NGAL)), an important component of the acute-phase reaction, increasingly is expressed in neutrophil granulocytes infiltrating the tissue, but also in hepatocytes activated by proinflammatory stimuli. Although neither hepatic mRNA levels nor protein expression of *Lcn2*/LCN2 was altered significantly, an increased infiltration of LCN2-positive cells as a characteristic feature of the biliary inflammatory process was shown by immunohistochemistry in the untreated *Abcb4*^*-/-*^ group (Figure 4*C–F*). Interestingly *Lcn2* was increased in the ACEA group in comparison with WT mice, but normalized to WT levels in rimonabant-treated mice (Figure 4*C*). The same effects were observed for the infiltration of LCN2-positive cells (Figure 4*F*). Moreover, rimonabant reduced the number of infiltrating LCN2-positive cells significantly (Figure 4*F*).

**Figure 5:**
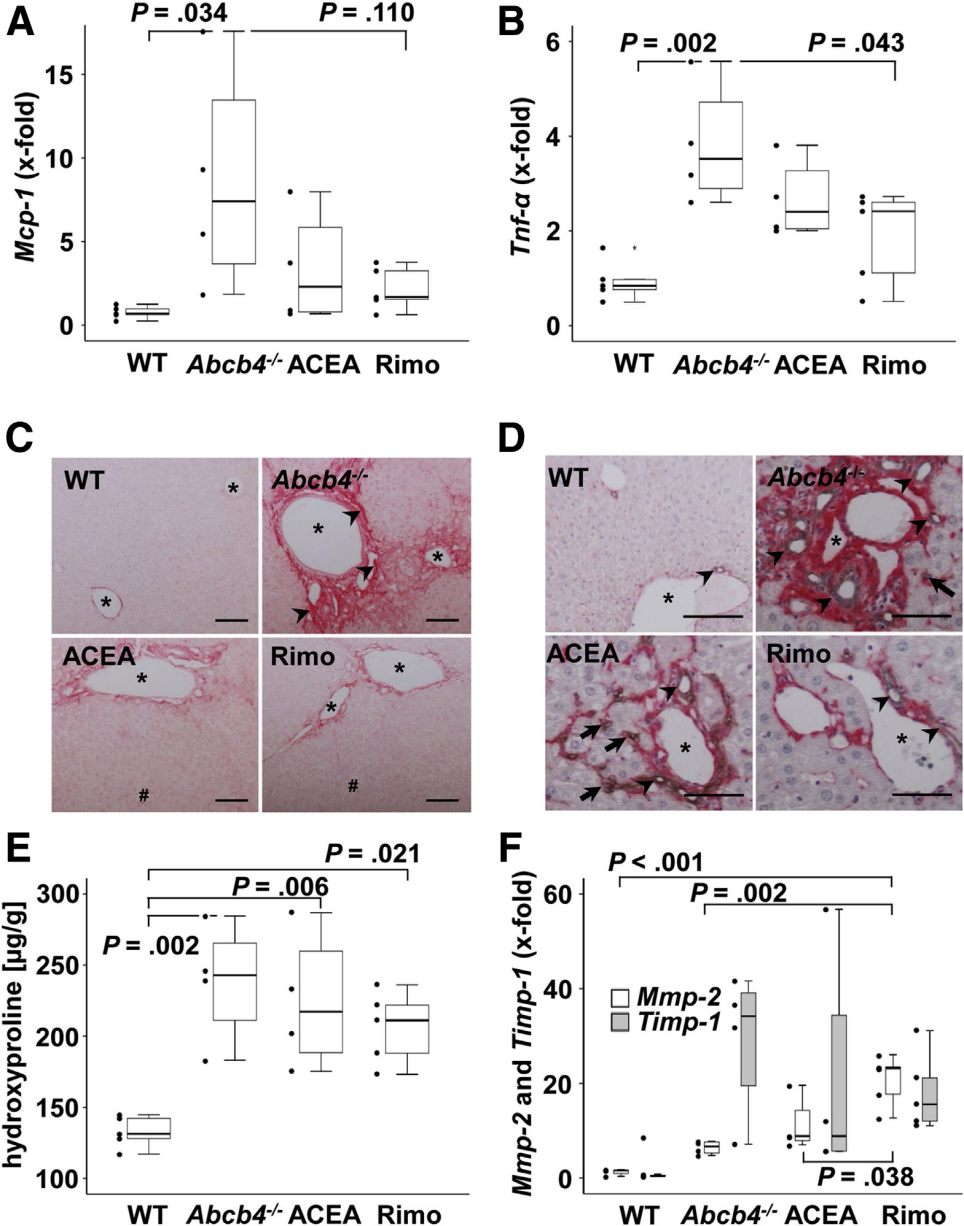
**ACEA and rimonabant did not alter hepatic fibrosis.** (*A* and *B*) Hepatic mRNA levels of (*A*) *Mcp-1* and (*B*) *Tnf-α* were enhanced in *Abcb4*^-/-^ mice. Treatment with rimonabant significantly reduced Tnf-*α*, whereas Mcp-1 was normalized to WT levels in tendency. (*C*) Sirius Red staining and (*D*) immunohistochemical costaining of type I collagen (red) and CK19 (grey) indicated reduced periportal fibrosis and ductular reaction by treatment with ACEA and rimonabant. Original magnification: (*C*) 100×, and (*D*) 200×. *Arrowheads* indicate collagen accumulation, *arrows* indicate bile ducts. ∗ marks portal vessels. (*C* and *D*) *Scalebars*: 100 μm. (*E*) Hydroxyproline quantification showed enhanced hepatic fibrosis in Abcb4^-/-^ mice as well as in ACEA-treated mice and moderate induction of fibrosis in rimonabant-treated mice. n = 4–5 mice per group. The experiment was performed 2 times. (*F*) Remarkably, transcriptional levels of the fibrosis marker *Timp-1* were not altered significantly among the groups although the hepatic expression of *Mmp-2* was induced in rimonabant-treated mice. (*A*, *B*, *E*, and *F*) One-way analysis of variance and post hoc Bonferroni tests were used for statistical analysis. n = 3–5 mice per group. One of 3 independent experiments of mRNA analyses is shown. Rimo, rimonabant.

Monocyte chemotactic protein 1 (*Mcp-1*), an inflammatory cytokine, recruits monocytes, T cells, and dendritic cells to the site of inflammation. Untreated *Abcb4*^*-/-*^ mice showed up-regulation of *Mcp-1*. Rimonabant reduced *Mcp-1* to WT levels (Figure 5*A*). Tumor necrosis factor-α has various functions in liver disease, including attraction and activation of inflammatory cells as well as mediation of hepatotoxicity and regeneration. *Tnf-α* was induced in untreated *Abcb4*^*-/-*^ mice and reduced to WT levels by rimonabant, while ACEA had no effect (Figure 5*B*).

### Fibrosis

Collagen-1 is the main component of fibrotic tissue. Sirius Red staining indicated pronounced fibrosis in untreated *Abcb4*^*-/-*^ mice with deposition of fibrillar collagen around the portal fields and around proliferating bile ducts (Figure 5*A*). Histologically, rimonabant-treated mice showed a lower collagen deposition, approximately at WT level, compared with untreated *Abcb4*^*-/-*^ mice. In addition, ACEA-treated mice showed increased hepatic collagen deposition, however, this was still lower than the level of collagen expression of untreated *Abcb4*^*-/-*^ mice (Figure 5*C*).

To analyze the locoregional correlation of collagen-1 and proliferating bile ducts, collagen-1/CK19-co-staining was performed (Figure 5*D*). We observed a spatial coherence of proliferating bile ducts and collagen-1 deposits in the untreated *Abcb4*^-/-^ group. *Abcb4*^*-/-*^ mice showed a strong deposition of collagen-1 and increased proliferation of bile ducts (arrowheads, Figure 5*D*). Moderate bile duct proliferation and collagen-1 deposition were observed in the rimonabant group, comparable with the WT group. Bile duct proliferation and collagen-1 deposition in ACEA-treated mice were similar to untreated *Abcb4*^*-*/-^ mice. The CK19-positive cells that did not form bile ducts appeared in the periphery of portal tracts in *Abcb4*^*-/-*^ and ACEA mice (arrows, Figure 5*D* *Abcb4*^*-/-*^ and ACEA). Nevertheless, it actually remains speculative whether these cells indicate the initiation of newly forming bile ducts or other associated processes (Figure 5*D*).

The cholestasis-dependent increase of hepatic hydroxyproline content in *Abcb4*^*-/-*^ mice appeared slightly reduced by ACEA and rimonabant treatment, but did not reach statistical significance (Figure 5*E*).

Fibrosis-associated genes such as *Timp1* and *Mmp2* were subjected to qRT-PCR analysis.

To analyze the proteolytic potential of extracellular matrix (ECM), the gene expression of *Mmp2* was analyzed by qRT-PCR. Treatment with rimonabant induced *Mmp-2* gene expression in comparison with WT and untreated *Abcb4*^-/-^ mice, while *Timp-1* mRNA levels were similar among all groups (Figure 5*F*).

Taken together, fibrosis was clearly induced in *Abcb4*^*-/-*^ mice whereas fibrogenesis did not appear to a lesser extend in rimonabant-treated mice. Nevertheless, histopathologic assessment of fibrosis was improved after rimonabant treatment. Interestingly, we observed a pronounced proteolytic potential indicated by *Mmp-2* induction in rimonabant-treated mice.

### Malignancy-Associated Signaling and Proliferation

Cellular-JUN and signal transducer and activator of transcription 3 (STAT3) are critical regulators of liver cancer development and progression.^14^^,^^15^ Both pathways also are involved in cholestasis-associated carcinogenesis.^16^ To analyze the activation of the c-Jun N-terminal kinase (JNK)/c-JUN signaling pathway, the phosphorylation of c-JUN was examined by Western blot in comparison with the expression of its unphosphorylated form (Figure 5*A*).

c-JUN was activated in liver tissue of *Abcb4*^*-/-*^ mice while rimonabant treatment resulted in reduced phosphorylation of c-JUN (Figure 5*A*), and reduced hepatocellular nuclear translocation (Figure 5*B*). STAT3 phosphorylation was not altered significantly between the mouse groups (Figure 5*C*). According to the regulatory role of c-JUN in the cell cycle,^17^^,^^18^ *cyclin D1* expression was reduced to WT levels by treatment with rimonabant (Figure 5*D*).

In summary, our results show that c-JUN was activated by chronic cholestatic liver damage in *Abcb4* knockout mice. The treatment with rimonabant reduced this activation to WT levels, while ACEA had no significant influence. Furthermore, c-JUN–associated proliferation, shown here by *Ccnd1* expression, followed the same trend.

## Discussion

The common final outcome of chronic liver diseases is the development of liver inflammation, fibrosis, and cirrhosis.^19^ Every year, an estimated 1,200,000 people worldwide die from its complications, including portal hypertension and hepatocellular carcinoma, thus demonstrating the need to establish new and effective treatment options for chronic liver diseases.^20^ The removal of the cause of liver injury can lead to regeneration of the damaged liver even with advanced cirrhosis.^21^ If this is not possible, there is still no effective antifibrotic or anticirrhotic therapy available to date.

Our present work has shown that the administration of the CB1 antagonist rimonabant in *Abcb4*^*-/-*^ mice did the following: (1) reduced damage, inflammation, and histopathologic fibrosis of the liver; (2) maintained liver integrity and zonation; and (3) reduced the activation of carcinogenesis-associated signaling pathways.

Thus, modulating the liver endocannabinoid system might be a potential therapeutic option to treat liver injury associated with cholestasis.

In different mouse models such as CCl_4_-, thioacetamide-, and bile duct ligation–induced fibrosis in resected human cirrhotic livers, in cell cultures of human hepatic stellate cells, and hepatic myofibroblasts, the stimulation of CB1 induced profibrotic effects, while the stimulation of CB2 resulted in the opposite outcome.^2^^,^^22^^,^^23^ In diet-induced obese mice, rimonabant had positive effects on liver metabolism and induced a reduction of liver fibrosis.^2^^,^^6^^,^^24^^,^^25^

Here, the influence of a pharmacologic modulation of CB1 by the CB1 antagonist rimonabant and ACEA (a CB1 agonist) in *Abcb4*^*-/-*^ mice on a Bagg Albino Mouse/c genetic background^26^ was examined. Up-regulation of SREBP-1c and *Fasn* in the signal cascade of activated CB1 contributes to the development of obesity and fatty liver via increased lipogenesis.^11^ In bile duct–ligated mice, the CB1 receptor is down-regulated during cholestasis.^8^ Accordingly, in *Abcb4*^*-/-*^ mice lipogenesis is reduced during cholestasis^13^ and these alterations in lipid metabolism mediate inflammation, fibrosis, and proliferation.^12^ In our study, we showed an increase of nuclear matured SREBP-1 in untreated *Abcb4*^*-/-*^ mice. Neither ACEA- nor rimonabant-treated *Abcb4*^*-/-*^ mice showed significant alterations of nuclear SREBP-1.

### Damage Caused by Cholestasis

In our study, increased ALT and AST values in untreated *Abcb4*^*-/-*^ mice showed increased liver cell damage, which did not occur in rimonabant-treated (and ACEA-treated) mice. The ALT values were reduced significantly under rimonabant, but still higher than the reference values of healthy WT mice (50 U/L).^27^ Incomplete ALT normalization could be attributed to the blood concentration of rimonabant being too low owing to the oral dosage form, incomplete absorption in the intestine, or any further metabolization.^23^^,^^28^ Nevertheless, an optimized pharmacologic antagonization of CB1 might be a promising target to handle liver damage in cholestasis.

The reduction in ALT level in the ACEA group was interesting because, in analogy to other models of chronic liver diseases, the values were expected to worsen.^29^ Repeated administration of CB1 agonists, however, could induce CB1 internalization or a reduction of CB1 protein synthesis.^30^ Coupling of ACEA to other receptors with protective effects on liver damage was unlikely for a long time because of its previously assumed high specificity for CB1. However, it could be shown that ACEA acts as an agonist of the transient receptor potential vanilloid 1.^31^ Activation of transient receptor potential vanilloid 1 by, for example, capsaicin, led to lower lipid droplet formation in the liver of high-fat diet–fed mice.^32^ Taking this into account, we speculate that ACEA administration may have a protective effect on some cholestasis-associated liver changes via the agonism at transient receptor potential vanilloid 1, which could explain some of the coherent effects by rimonabant and ACEA that we describe with the current study.

Because of an increased permeability of bile duct epithelium, increased bile acid concentrations can be measured in the blood circulation of *Abcb4*^*-/-*^ mice.^33^ The conjugation of bile acids with amino acids such as taurine or glycine increases their detergent properties and prevents their precipitation in an acidic environment. It is known that the bile acids in rodents are 80%–90% taurine-conjugated.^34^^,^^35^ Lower concentration levels of total bile acids in the serum were measured after ACEA and rimonabant treatment compared with the untreated group. Missing statistical significance might be owing to the limited group size and high number of different bile acids measured. Interestingly, ACEA-treated mice reached lower levels than rimonabant-treated mice with regard to inflammation and bile acid concentration. In the analysis of bile acids in the serum, the dominance of taurine-conjugated bile acids, in particular, the tauro-β-muricholic acid, tauro-α-muricholic acid, and tauro-ω-muricholic acid, was confirmed in all samples.^9^

The reduced parenchymal damage (Figure 1*B* and *C*) indicates that the agonization of the CB1 receptor also might have hepatoprotective effects in *Abcb4*^*-/-*^ mice. As described earlier, this may be owing to further interactions of ACEA on hepatic receptors. However, the protective effect of rimonabant in *Abcb4*^*-/-*^ mice is congruent with other models of chronic liver damage such as CCl_4_-induced, thioacetamide-induced, and bile duct ligation–induced liver fibrosis.^2^

### CB1 in View of Hepatic Glucose and Lipid Metabolism

In the context of cholestatic diseases, the liver parenchyma and thus the liver architecture and the sophisticated zonation is disrupted. In the present work, an abolished zonation in untreated *Abcb4*^*-/-*^ mice as well as in ACEA-treated *Abcb4*^*-/-*^ mice could be shown by immunohistochemical staining for PCK1 and FASN (Figure 3*A* and *E*). Physiologic zonation was regained by treatment with rimonabant.

The endocannabinoid system contributes to the development of steatosis, dyslipidemia, and insulin resistance.^36^ PCK1, the rate-determining enzyme of gluconeogenesis, did not show clear staining in the periportal zone, but a diffuse distribution of PCK1-positive hepatocytes.^37^ Ghafoory et al^38^ showed that the CCl_4_-induced liver damage led to a strong change in the gene expression for enzymes of glucose metabolism in the periportal and perivenous zones. The typical zonal expression of PCK1 was largely preserved through rimonabant treatment (Figure 3*A*), which also reflects serious protective effects of rimonabant on metabolism. The gene expression levels of *Pck1* did not show any differences between the groups (Figure 2*C*), and serum glucose concentrations were within the normal range in all groups. Thus, euglycemia probably might be attributed to a compensated metabolism in the stage of liver fibrosis, but not yet cirrhosis or decompensation.

Various models have shown that hepatic CB1 activation increases de novo lipogenesis by activating *Srebp1c* and *Fasn*, and at the same time reduces fatty acid oxidation.^11^^,^^39^ Analogous to a previous study,^11^ untreated *Abcb4*^*-/-*^ mice showed down-regulation of *Fasn* expression in our study. The normalization of *Fasn* expression by rimonabant was associated with a normalization of metabolic processes. Immunohistochemistry for FASN indicated a diffuse distribution of FASN-positive hepatocytes in untreated *Abcb4*^*-/-*^ mice and a normalization of the typical zonation in rimonabant-treated mice.

Recently, we showed that the CB1 knockout in vivo and pharmacologic antagonization of CB1 in cell culture decreased PLIN2 expression, which might be an essential step in lipid breakdown.^7^ In this study, we show that the pharmacologic modulation of CB1 represents a novel therapeutic approach for the treatment of cholestatic liver injury.

### Regulation by PPARs

In the liver, a protective effect of PPARα on the development of NASH and inflammation was shown by down-regulation of nuclear factor kappa-light-chain-enhancer of activated B cells, activator protein 1, STATs, and interleukin 6.^40^ In the present work, *Pparα* was tendentially reduced in untreated *Abcb4*^*-/-*^ mice compared with WT. In the rimonabant group, normalization of *Pparα* to WT level was observed, while *Pparα* expression remained unchanged with ACEA treatment (Figure 3*B*). Because there were no differences in the serum glucose concentrations, the cholestatically reduced *Pparα* expression appears to have no effect on stable hepatic glucose metabolism. Among others, PPARγ regulates the differentiation of adipocytes and contributes to lipid accumulation in the liver. These effects are moderated by induction of SREBP-1c, acetyl-CoA carboxylase (ACC), and FASN.^41^ Interestingly, *Pparγ* expression was induced significantly in the rimonabant group. The ACEA group, however, showed no differences compared with the untreated *Abcb4*^*-/-*^ mice.

### Hepatic Inflammation

The enhanced number of infiltrated CD45^+^ leukocytes in *Abcb4*^*-/-*^ mice was abolished by rimonabant, which indicates an anti-inflammatory effect of pharmacologic CB1 antagonization during cholestasis. Up-regulation of lipocalin 2 during endoplasmic reticulum (ER) stress-induced inflammatory responses protects hepatocytes from being overwhelmed by unfolded protein response upon liver injury.^42^

On the other side, LCN2 is secreted into the serum from liver cancer tissue in human beings and mice.^43^ *Lcn2* increasingly was expressed in the ACEA group, whereas the rimonabant group showed normalized *Lcn2* expression on the WT level. A clinical study of 716 patients with cirrhosis showed that LCN2 might be a biomarker of acute-on-chronic liver failure and prognosis in cirrhosis.^44^^,^^45^ Because LCN2 is a good candidate for hepatocellular carcinoma diagnosis and screening, the reduction of *Lcn2* might indicate a beneficial development. Interestingly, the untreated group and the ACEA group did not show up-regulation of interleukin 6, nuclear factor kappa-light-chain-enhancer of activated B cells, I-κB, protein kinase B, or other mediators inducing the aforementioned LCN2 (data not shown). It must be assumed that LCN2 is activated by the damaged hepatocytes via alternative routes. The measurement of proinflammatory markers such as LCN2, tumor necrosis factor-α, and monocyte chemoattractant protein-1 shows the anti-inflammatory effect of rimonabant, which correlated with reduced liver damage in our murine model.

### Fibrosis

As expected, untreated *Abcb4*^*-/-*^ mice showed an increased overall hepatic collagen deposition, which was not altered by treatment with ACEA or rimonabant. Nevertheless, histopathologic assessment of fibrosis was improved after rimonabant treatment. The net deposition of scar tissue depends on the balance between synthesis and degradation.^3^^,^^46^ The latter reflecting the relative activity of matrix metalloproteinases and their tissue inhibitors of metalloproteinases, which are produced primarily by hepatic stellate cells. The activation of matrix metalloproteinases leads to the dissolution of the deposition of ECM. The activity of the matrix metalloproteinases thus leads to fibrosis regression.^46^^,^^47^ On gene expression levels, the treatment with rimonabant led to an up-regulation of the proteolytic potential, enabling enhanced degradation of ECM in the fibrotic liver. This fact may be reflected by the moderate level of fibrosis in rimonabant-treated animals.

### Signal Transduction

**Figure 6:**
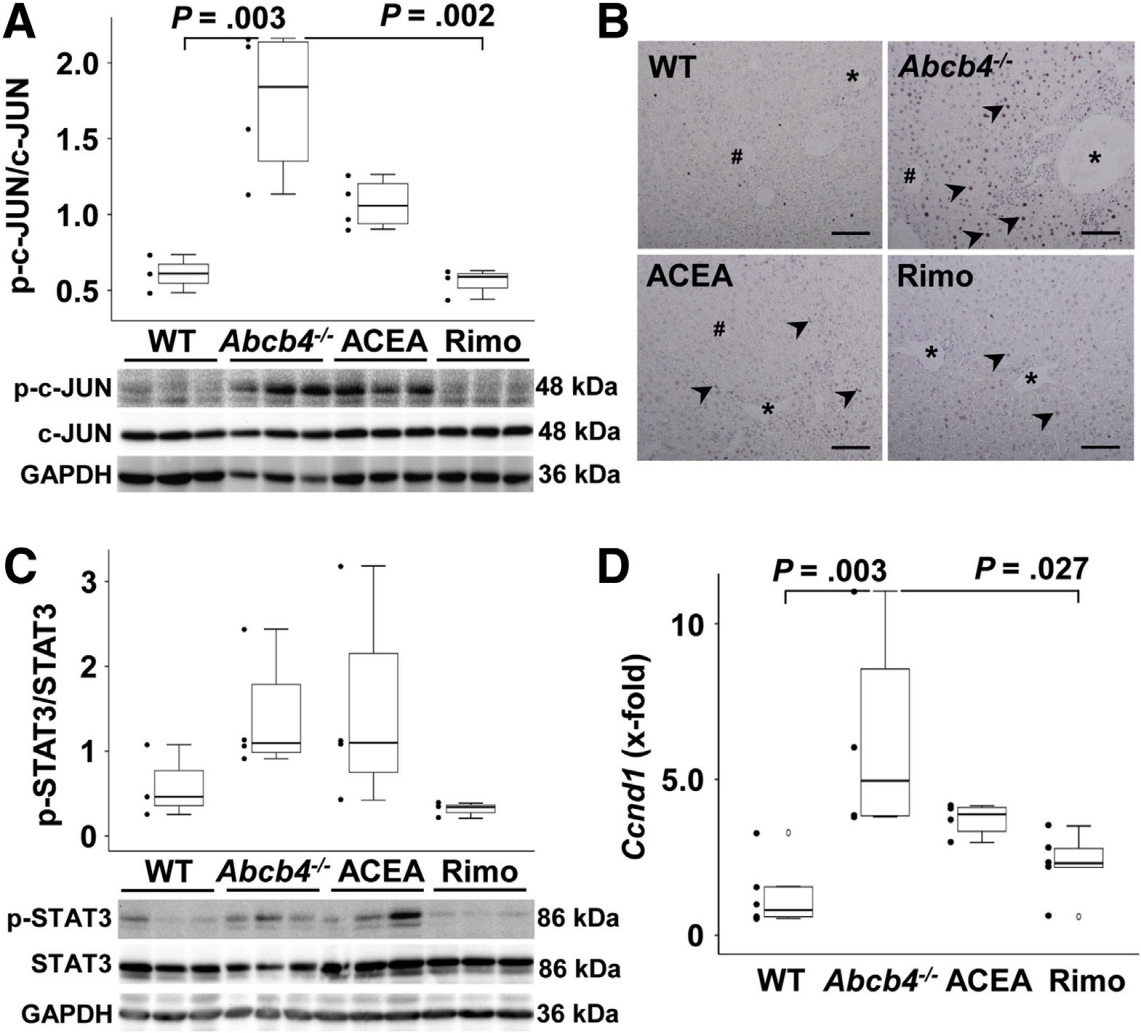
**Rimonabant reduced the activation of c-JUN signaling as well as *Ccnd1* in *Abcb4***^**-/-**^**mice.** (*A*) Western blot analysis showed the activation of c-Jun in *Abcb4*^-/-^ mice and the reduction of hepatic c-JUN activation by rimonabant. (*B*) Immunohistochemistry showed more c-JUN–positive nuclei in hepatocytes of *Abcb4*^-/-^ mice and a decrease of hepatocellular c-JUN activation (*arrowheads*) by treatment with rimonabant. ∗Portal vessels, #central veins. (*C*) The level of activation was not altered significantly among the groups. (*D*) Cholestasis-induced induction of hepatic *Ccnd1* transcription in *Abcb4*^-/-^ mice was normalized to WT level by rimonabant. (*A*, *C*, and *D*) One-way analysis of variance and post hoc Bonferroni tests were used for statistical analysis. n = 3–5 mice per group. One of 3 independent experiments of mRNA analyses is shown. For protein expression, representative data of 3 independent experiments are depicted. GAPDH, glyceraldehyde-3-phosphate dehydrogenase; p-c-JUN, phosho-cellular JUN; p-STAT3, phosho-signal transducer and activator of transcription 3; Rimo, rimonabant.

In acute and chronic liver damage, JNK1 and its downstream signals are activated and contribute to disease progression.^48^ In the present work, Western blot analysis of untreated *Abcb4*^*-/-*^ mice showed an increased activation of c-JUN. The hepatoprotective effect of rimonabant treatment was reflected in a lower activation of c-JUN, which was normalized to WT level. In 2001, Gupta et al^49^ already showed a direct activation of JNK1 by taurine-conjugated bile acids in a model of rat hepatocytes. C-JUN and *cyclin D1* were activated in untreated *Abcb4*^*-/-*^ mice (Figure 6). Rimonabant reduced this activation to WT level. Treatment with ACEA had no effect.

### Limitations

A limitation of the study was the way in which medication was administered via the drinking water, which means that different concentrations of active substances cannot be ruled out on an individual basis. Both the amount of substances absorbed via the intestine and the further metabolization remain unknown factors. Alternatives such as intravenous or intra-abdominal injection did not appear physiological. In addition, these procedures might be technically difficult, associated with animal stress, and time consuming.

In summary, the CB1 blockade in *Abcb4*^*-/-*^ mice by rimonabant enabled a reduction of liver damage as well as inflammatory and acute phase markers such as LCN2, *Mcp1*, and *Tnf-α*. With regard to liver metabolism, treatment with rimonabant resulted in the preservation of the typical liver zonation. Proliferation and carcinogenesis-associated signaling pathways were normalized to WT levels by treatment with rimonabant. We thus conclude that the regulation of the JNK signaling pathway by CB1 modulation might play an important role in cholestatic liver diseases and, if applicable, in hepatic carcinogenesis.

## Materials and Methods

### Animal Treatment

The present study was performed with permission of the State of Hesse, Regierungspraesidium Giessen, according to section 8 of the German Law for the protection of animals, and conforms to the National Institutes of Health guide for the care and use of laboratory animals. All experiments were approved by the committee on the ethics of animal experiments of the Regierungspraesidium Giessen, Germany (permit number: V54-19c2015 (1) GI20/10 no. 52/2011). Bagg Albino Mouse/c-*Abcb4*^*-/-*^ mice (C.FVB[129P2]-*Abcb4*^tm1Bor^, herein referred to as *Abcb4*^*-/-*^ mice) were bred and housed as described previously.^26^ Characterization of *Abcb4*^*-/-*^ genotype, sample collection, and routine analysis have been described elsewhere.^26^ In this project, the population of male *Abcb4*^*-/-*^ knockout mice was divided into 3 groups. One group (n = 4) remained untreated and received standard chow. The second group (n = 4) was treated with 1 mg/kg body weight per day ACEA after weaning from the mother in the third week of life. A third group (n = 5) was fed with 1 mg/kg body weight per day rimonabant. Standard chow (R/M-H) supplemented with ACEA and rimonabant was obtained from Sniff (59494; Spezialdiäten GmbH, Soest, Germany). Twenty kilograms of chow was charged with 133 mg rimonabant or 125 mg ACEA.

Mice were killed at the age of 16 weeks. Livers and blood sera were isolated and stored at -80°C. Sixteen-week-old WT mice were used as healthy supercontrols.

### Histology and Immunohistochemistry

Preparation of 3-μm paraffin sections, H&E staining, Sirius red staining, immunohistochemistry, microphotography, and scoring was performed as described before.^50^ The following specific primary antibodies were used for immunohistochemistry: CK19 (ab15463-1; Abcam, Boston, MA), PCK1 homemade (Bruno Christ) antibody was raised in rabbits using a synthetic peptide comprising amino acids 385–399 of the cytosolic form of phosphoenolpyruvate carboxykinase, FASN CST 3189, CD45 CST 70257, LCN2 (sc-80234; Santa Cruz, TX), type I collagen sc-33111 (Santa Cruz, TX), and pc-Jun 3270 (Cell Signaling, Frankfurt, Germany). Unspecific isotype IgGs were used for control.

### Bile Acid Analysis

Bile acids were quantified by Ultra performance liquid chromatography–tandem mass spectrometry, as has been described in the literature.^51^

### Hydroxyproline Assay

The total hepatic hydroxyproline content was quantified as described previously with minor modifications.^52^^,^^53^ Briefly, 50 mg mouse liver tissue was hydrolyzed in 1 mL 6 N HCl at 110°C for 14 hours. Hydrolysates were filtered through 45-μm pore filters (Sartorius, Göttingen, Germany). A total of 15 μL of the hydrolysate was dried under nitrogen flow and subsequently redissolved in 50 μL 50% 2-propanol. A total of 100 μL of 0.6% chloramine-T (Merck, Darmstadt, Germany) solution was added to the samples and hydroxyproline standard probes (4-hydroxy-L-proline; Sigma-Aldrich, Taufkirchen, Germany) and incubated for 10 minutes at room temperature. Ehrlich’s solution (100 μL, 3 g dimethylamino-benzaldehyde [Sigma-Aldrich] in 26 mL 2-propanol + 8 mL 70% perchloric acid) was added and the samples again were incubated for 45 minutes at 50°C. Absorbance was measured at 570 nm using a microplate reader (Packard BioScience, Meriden, CT). Hydroxyproline levels were calculated against standard curves and expressed as milligrams of hydroxyproline per gram of liver tissue.

### Quantitative Real-Time PCR

RNA extraction and complementary DNA synthesis as well as qRT-PCR were performed as described previously.^50^ Briefly, hepatic RNA was extracted using the RNeasy Mini (QIAGEN, Hilden, Germany), and elimination of genomic DNA was performed with the TURBO DNAfree-Kit (Thermo Fisher Scientific, Waltham, MA) according to the manufacturer’s instructions. RNA integrity and purity were analyzed by gel electrophoresis and spectrophotometry and equal amounts of RNA were transcribed into complementary DNA using the iScript complementary DNA Synthesis-Kit (Bio-Rad, Hercules, CA). qPCR was performed using a StepOnePlus real-time PCR system (Life Technologies, Darmstadt, Germany) and SYBR-Green/ROX dye (Sigma Aldrich, Steinheim, Germany). Primers were purchased by Microsynth (Göttingen, Germany). Individual gene expression was calculated according to the delta delta cycle treshold (ΔΔCt) method.^54^

### Western Blot

Western blot experiments were performed as described before^55^ using antibodies against SREBP1 (bs-1402R; BIOSS, Woburn, MA), LCN2 (AF1857; R&D Systems, Abingdon, UK), as well as phosho-c-JUN (3270), c-JUN (9165), p-STAT3 (Signal Transducers and Activators of Transcription 3) (9145), and STAT3 (4904P), all purchased from Cell Signaling Technology, Inc (Danvers, MA). Mouse anti–β-actin monoclonal antibodies (sc-47778; Santa Cruz Biotechnology, Inc, Dallas, TX) or Ponceau C–stained blots were used for loading controls. Semiquantitative analysis of obtained signals was performed using ImageJ software (National Institutes of Health, Bethesda, MD).^56^

### Triglyceride Measurement

Triglyceride quantification was performed according to the manufacturer’s instructions (ab65336; Abcam, Cambridge, MA).

### Statistics

The data were processed and analyzed with IBM SPSS Statistics version 26.0. (NewYork, NY) The distribution of the residuals was checked with graphic methods (QQ plot) and no significant deviation from the normal distribution was found. All parameters were analyzed with a 1-way analysis of variance test and a post hoc Bonferroni test. Bonferroni corrected significance levels are presented.

All authors had access to the study data and reviewed and approved the final manuscript.
